# Feasibility Evaluation of Detecting Hydroxymethylphosphine Oxide *In Vivo* by ^31^P-MRS

**Published:** 2010-09

**Authors:** Sabrina Doblas, Gopal Pathuri, Rheal A. Towner, Hariprasad Gali

**Affiliations:** 1 *Advanced Magnetic Resonance Center, Oklahoma Medical Research Foundation, 825 NE 13th street, Oklahoma City, Oklahoma, USA;*; 2 *Department of Pharmaceutical Sciences, The University of Oklahoma College of Pharmacy, 1110 N. Stonewall Avenue, Oklahoma City, Oklahoma, USA*

**Keywords:** ^31^P-MRS, hydroxymethylphosphine oxide, MRI, NMR

## Abstract

Application of organophosphorus compounds in biomedicine is attractive because the ^31^P nucleus is very amenable to study by nuclear magnetic resonance (NMR) techniques, particularly, by *in vivo* ^31^P magnetic resonance spectroscopy (^31^P-MRS). The water-soluble organophosphorus compounds that are non-toxic, exhibit metabolic stability, and show a unique resonance peak in ^31^P NMR spectroscopy, which could be ideal to be used as probes for ^31^P-MRS. Here we evaluated the *in vivo* feasibility of potentially using a hydroxymethylphosphine oxide as a novel probe for ^31^P-MRS studies using tris (hydroxymethyl) phosphine oxide (THPO) as an example. THPO was synthesized, injected in the normal CF1 mice, and ^31^P spectra were acquired before and after injection with the coil located on the regions of interest. The NMR signal from the region of interest appeared within one minute of THPO injection. The compound was stable *in vivo* as no metabolites of THPO were observed. No toxicity was observed after THPO injection in mice. The peak concentrations of THPO in liver and kidney were reached within 15 min and 60 min respectively. THPO was excreted exclusively in urine without undergoing any metabolism indicating that it is very stable under *in vivo* conditions. These initial studies in normal CF1 mice clearly demonstrate that THPO possess the essential characteristics required for a potential MRS probe. Based on the current preliminary results, we suggest that HMPs, when incorporated into targeted drugs (peptides, proteins, antibodies, *etc*.), may serve as novel ^31^P probes for monitoring the drug distribution *in vivo* by MRS.

## BACKGROUND

Application of organophosphorus compounds in biomedicine is attractive because the ^31^P nucleus is very amenable to study by nuclear magnetic resonance (NMR) techniques, particularly, by *in vivo* ^31^P magnetic resonance spectroscopy (^31^P-MRS). The advantages of using ^31^P-MRS are: a) ^31^P is a desirable NMR nucleus with 100% natural abundance, high NMR sensitivity (~6.6% of ^1^H), relatively large g value (10,830 rad/gauss), and a large chemical shift range (1); b) the technique is intrinsically noninvasive; c) there is no radioactivity involved and therefore the compounds are inherently stable with a long shelf life, allowing studies at later time points after injection when the blood background is diminished; and d) it is possible to obtain high-resolution anatomic ^1^H magnetic resonance imaging (MRI) in conjunction with the ^31^P NMR spectra, which provide context in terms of organs and tissue heterogeneity, with no need for imaging after processing and co-registration procedures.

The organophosphorus compounds that are non-toxic, soluble in biologically compatible solvents, exhibit metabolic stability, and show a unique resonance peak in ^31^P NMR spectroscopy, are ideal to be used as probes for ^31^P-MRS. Here we discuss the utility of a tris (hydroxymethyl) phosphine oxide (THPO) as a probe for ^31^P MRS. Little is known regarding the toxicity of THPO. THPO was not found to be mutagenic in Salmonella and did not inhibit acetyl cholinesterase *in vitro* (2, 3). THPO was nominated by the National Toxicology Program in 2005 to undergo toxicity tests, since it is a by-product and metabolite of tetrakis (hydroxymethyl) phosphonium chloride (THPC), a reactive flame retardant widely used with cellulosic and cellulosic blend fabrics, including FR-treated infant sleepwear and work clothes (4).

Katti *et al*. have reported the utility of hydroxymethylphosphines as chelating ligands for labeling biomolecules with ^99m^Tc/^188^Re for developing targeted diagnostic/therapeutic radiopharmaceuticals (5-7). However, no organophosphorus compound has ever been utilized for any other diagnostic application. To our knowledge, this is the first report that describes the utility of an organophosphorus compound as an extrinsic ^31^P-MRS probe. ^31^P-MRS is currently used to determine intrinsic phosphorus metabolite concentrations in human tissues such as muscle and brain (8, 9).

## METHODS

### Materials

Tetrakis (hydroxymethyl) phosphonium chloride, triethylamine, and 30 wt% hydrogen peroxide were purchased from Sigma-Aldrich (St. Louis, MO). Tris (hydroxymethyl) phosphine (THP) was synthesized by slight modifications to the previously reported procedure (10). ^31^P NMR spectroscopy was performed on a Varian Mercury VX-300 NMR Spectrometer at the NMR Facility (University of Oklahoma, Norman, OK). CF-1 (19-21 g) male mice were purchased from Charles River Laboratories International, Inc. (Wilmington, MA). All animal studies were conducted in accordance with the protocols approved by both the OUHSC and OMRF institutional animal care and use committees. All MR experiments were conducted using a 2.0 cm surface coil in a 7 Tesla 30 cm horizontal bore small animal MRI system (Bruker BioSpin MRI, Germany) at the Advanced Magnetic Resonance Center at the Oklahoma Medical Research Foundation, Oklahoma City, OK.

### Tetrakis (hydroxymethyl) phosphine oxide (THPO)

To a solution of THP (3.4 g, 27.4 mmol) in water (6 ml) was added 30 wt% hydrogen peroxide in water (8.8 ml, 77.6 mmol). The reaction mixture was heated to 35 °C for 30 min. The progress of the reaction was monitored by ^31^P NMR. After completion of the reaction, the water was evaporated on rotary evaporator to obtain the THPO as a white solid (yield - 3.6 g, 93.8%). The THPO was used without any further purification. ^1^P NMR (D_2_O, 121.47 MHz) δ (ppm) 48.9 (s, 1P); ^13^C NMR (D_2_O, 75.45 MHz) δ (ppm) 52.2 (d, 3C).

## ^31^P MRS

### THPO frequency

To determine the frequency of the THPO, two 1.5 ml centrifuge tubes (one containing 1M phosphoric acid as a reference and the other containing 1M THPO solution) were placed at the isocenter of the magnet and a ^31^P single pulse spectrum was acquired with a repetition time (TR) of 3,000 ms, 2,048 points, an acquisition time of 146.23 ms and a spectral width of 14 kHz. The THPO frequency was determined to be 121.589 MHz.

### T1 relaxation time of THPO

To identify the T1 relaxation time of the THPO, a series of ^31^P RARE images with varying TR were acquired on a 1.5 ml centrifuge tube containing 1M THPO solution (scan parameters: echo time (TE)=14.4 ms; TR=250, 500, 750, 1,000, 1,500, 2,000, 2,500, 3,000, 4,000 and 5,000 ms; matrix size=32 × 32 pixels; field of view=8 × 8 cm^2^; 1 axial slice of 5 mm thickness). Regions of interest were drawn on the tube to get signal intensity measurements that were fitted to an equation linking signal intensity to TR:

$S=C_{1}-C_{2}e^{\frac{-\mathrm{TR}}{\mathrm{T1}}}$

The T1 of THPO was determined to be 2,990 ms. As a consequence, a TR of 3,000 ms was chosen for subsequent experiments.

### Animal experiments

Animal experiments were conducted at a frequency of 121.589 MHz using a ^31^P single pulse sequence over a spectral width of 8,000 Hz, 32 averages, a TR of 3,000 ms, 2,048 points, and an acquisition time of 1 min 36 s. A 72 mm-diameter ^1^H/^31^P resonator was used for signal transmission and the signal was received through a tunable ^1^H/^31^P flat surface coil positioned on the region of interest of the sample (phantom or mouse). Normal CF-1 mice were anesthetized initially using 2% isoflurane in oxygen at 2 L/min, in a polypropylene induction chamber. When fully anesthetized, a catheter was placed in the tail vein and the animals were placed on the scanner bed. Anesthesia was maintained through a nose cone with 1% isoflurane in oxygen at 2 L/min. Body temperature was maintained at 37 ± 1°C by using a water-circulated pad under the animal. Animals were placed in a supine position for the liver experiments, or on its side for the kidney experiments, and the ^1^H/^31^P surface coil was located on the organs of interest. A quick ^1^H-T_1_-weighted morphological image was acquired to guarantee the proper positioning of the surface coil, and a ^31^P “baseline” spectrum was acquired. 100 μl of 4M THPO solution was then administered through the tail vein, and ^31^P spectrum acquisition was resumed for 2 hours (liver experiment) or 4 hours (kidney experiment). Following MR experiments, the mice were euthanized, urine and blood of the animal were collected, and ^31^P spectra were acquired on these *ex vivo* samples. ^31^P spectra were pre-processed using the Bruker TopSpin software (apodization, Fourier transformation, and phasing of spectra), and then calibrated and integrated to provide peak areas using Mathematica (Wolfram Research).

## RESULTS AND DISCUSSION

**Figure 1:**
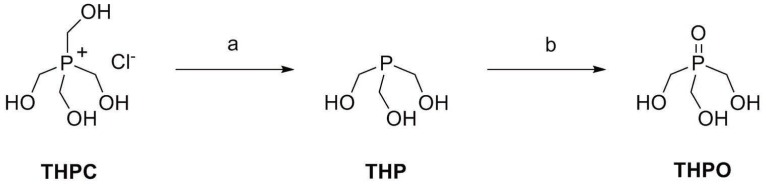
Synthesis of THPO. a) Triethylamine (10 eq), 8 h, room temp; b) 33 wt% H_2_O_2_ (2.8 eq), 0.5 h, 35 °C.

**Figure 2:**
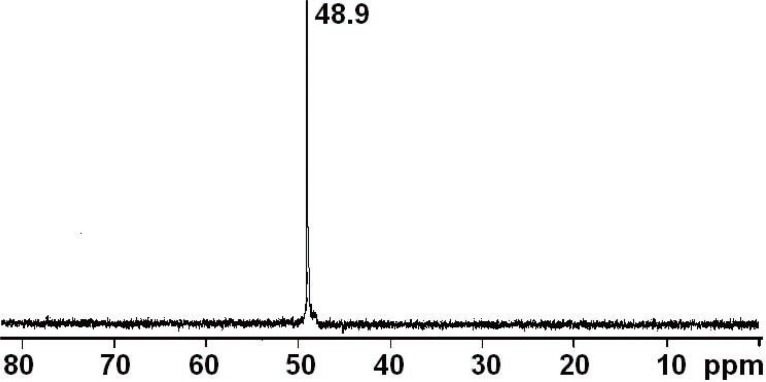
^31^P NMR spectrum of THPO.

To demonstrate the proof-of-concept, we synthesized THPO and evaluated the feasibility of observing this compound using ^31^P-MRS in normal CF1 mice. THPO was synthesized in a two-step reaction as shown in Figure 1. First, commercially available THPC was treated with triethylamine to obtain THP and then it was oxidized with hydrogen peroxide to obtain THPO. THPO is soluble in water at all concentrations due to the presence of hydrophilic -CH_2_OH substituents, and presents a single peak in ^31^P NMR spectra with a chemical shift of ~49 ppm as shown in Figure 2. This chemical shift is farther down field than that of all indigenous phosphorous containing compounds (<20 ppm) found in the body.

**Figure 3:**
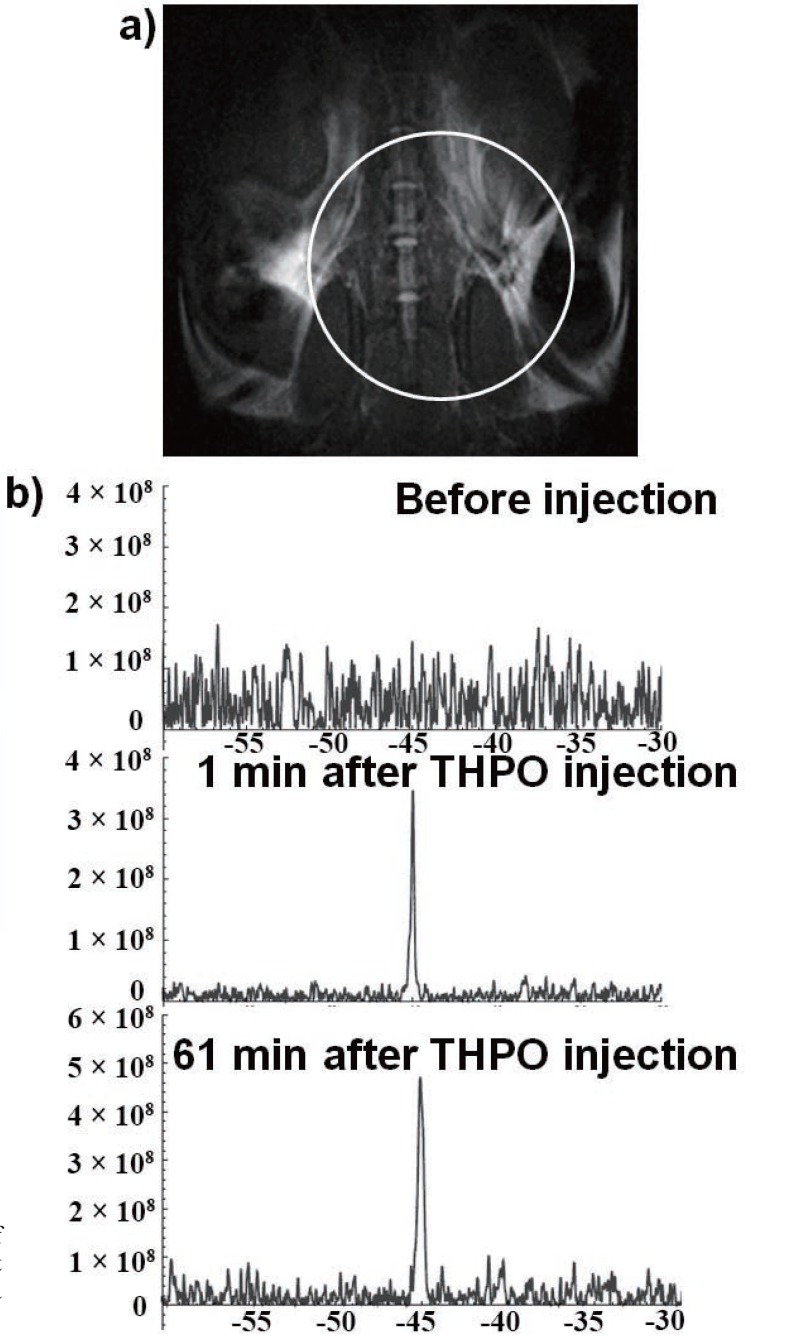
a) Representative ^1^H-MRI image of a mouse (circle shows the region of interest observed by the surface coil); b) ^31^P spectra obtained from the region of interest.

For *in vivo* evaluation, we injected THPO solution in the first mouse and acquired ^31^P spectra before and after injection with the coil located between the kidneys and the bladder, as shown in Figure 3. The NMR signal from the region covering the kidneys and the bladder appeared within one minute of injection. The compound was stable *in vivo* as no metabolites of THPO were observed. Only a single peak corresponding to THPO was observed throughout the imaging period of 61 min (Figure 3b). After THPO injection, the mouse behaved normally and no adverse reactions were observed.

**Figure 4:**
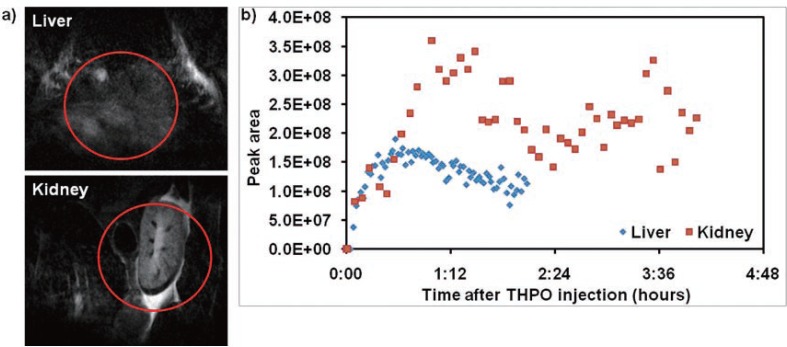
a) ^1^H-MRI image from a representative mouse (circle shows the surface coil region of interest); b) Peak areas of the THPO resonance in liver and kidneys are plotted as a function of time after injection in two different mice.

**Figure 5:**
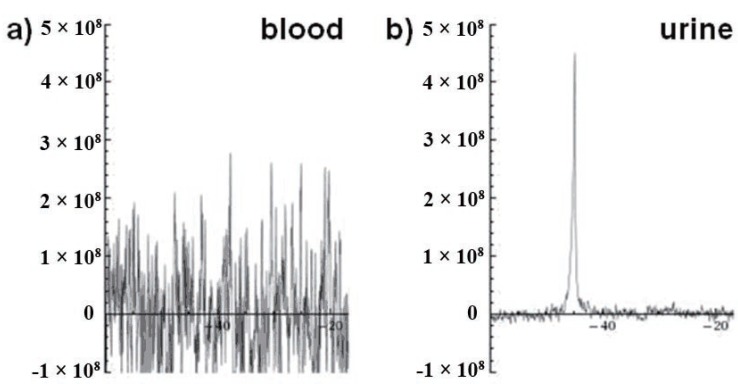
^31^P spectra of a) blood and b) urine collected from a mouse 2 hours after injection of THPO.

To follow the clearance of THPO from the blood pool, we injected two mice with THPO and assessed the liver in one mouse for 2 h, and the kidney in the other mouse for 4 h (Figure 4). The mice were killed, and the blood and urine were collected at the end of the imaging study. As shown in the Figure 4b, the peak concentrations in the liver and the kidneys were reached within 15 min and 60 min, respectively. Since THPO is very hydrophilic, as expected it was cleared predominantly through the kidneys into urine (Figure 5). The clearance from the kidneys was relatively slower because THPO is a small hydrophilic and neutral molecule that may be just cleared by glomerular filtration but not secreted by the tubules. As shown in Figure 5a, no THPO was observed in the blood samples collected from the mice indicating that THPO was completely cleared from the blood pool by 2 h. THPO was excreted exclusively in urine without undergoing any metabolism indicating that it is very stable under *in vivo* conditions (Figure 5b). More *in vivo* studies are needed to determine the pharmacokinetic properties of THPO, as well as toxicity. These initial studies in normal CF1 mice clearly demonstrate that THPO possess the essential characteristics required for a potential MRS probe.

**Figure 6:**
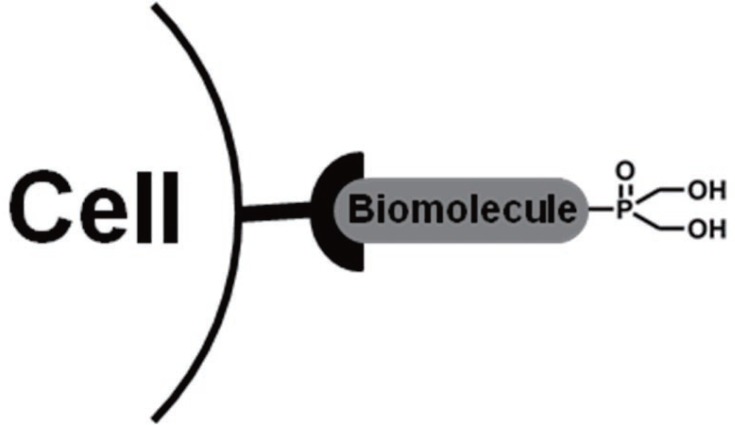
Design of a molecular-targeted ^31^P-MRS probe utilizing a hydroxymethylphosphine oxide.

New HMPO derivatives containing functional groups such as –COOH that allow conjugation to biomolecules (*e.g.*, peptides, proteins, antibodies, *etc.*), as shown in Figure 6, are needed to increase the concentration of the probe at the targeted disease site. The hydrophilic nature of HMPOs facilitates their efficient clearance from the blood pool primarily through the renal/urinary pathway if they are separated from biomolecules due to any *in vivo* degradation process. Thus, this minimizes the background signal due to presence of free HMPO in the blood pool. However, the biodistribution and pharmacokinetic properties of the HMPO-biomolecule conjugate may predominantly be dominated by those of the biomolecule itself.

Based on the current preliminary results, we suggest that HMPOs, when incorporated into targeted drugs (peptides, proteins, antibodies, *etc.*), may serve as novel ^31^P probes for monitoring the drug distribution *in vivo* by MRS. However, due to the inherent low sensitivity, it is a challenging endeavor to develop a molecular-targeting MRS probe. We will undertake further studies to address the critical issue of sensitivity with HMPO based molecular-targeting MRS probes in the future.

## ABBREVATIONS

HMPO: = Hydroxymethylphosphine oxide
THPO: = Tris (hydroxymethyl) phosphine oxide
THPC: = Tetrakis (hydroxymethyl) phosphonium chloride
THP: = Tris (hydroxymethyl) phosphine
MRS: = Magnetic resonance spectroscopy
MR: = Magnetic resonance
NMR: = Nuclear magnetic resonance
ppm: = Parts per million
TR: = Repetition time
TE: = Echo time
